# *ABCA12* Promotes Proliferation and Migration and Inhibits Apoptosis of Pancreatic Cancer Cells Through the AKT Signaling Pathway

**DOI:** 10.3389/fgene.2022.906326

**Published:** 2022-06-16

**Authors:** Songyuan Zheng, Dongyan Liu, Feifei Wang, Youyan Jin, Siqiao Zhao, Siyu Sun, Sheng Wang

**Affiliations:** ^1^ Department of Endoscopy Center, Shengjing Hospital of China Medical University, Shenyang, China; ^2^ Department of Gastroenterology and Medical Research Center, Liaoning Key Laboratory of Research and Application of Animal Models for Environmental and Metabolic Diseases, Shengjing Hospital of China Medical University, Shenyang, China

**Keywords:** pancreatic cancer, *ABCA12*, cancer, proliferation, migration, apoptosis

## Abstract

**Background:** As a malignant tumor, pancreatic cancer is difficult to detect in its early stage. Pancreatic cancer progresses rapidly and has a short survival time. Most cases have metastasized to distant organs before diagnosis. The mechanism of induction of pancreatic cancer is not fully understood.

**Methods:** In this study, bioinformatics predicted ATP binding cassette subfamily A member 12 (*ABCA12*) expression in pancreatic tissues and performed survival analysis, risk assessment, and enrichment analysis. The expression of *ABCA12* in 30 pairs of clinical samples was detected by immunohistochemistry and we analyzed its correlation with clinical information. Both reverse transcription polymerase chain reaction (RT–PCR) and western blot analysis were used to detect mRNA and protein expression in cell lines. Two different siRNAs and SW1990 cell line were used to construct pancreatic cancer cell models with *ABCA12* knockdown. Cell viability was evaluated by cell counting kit-8 (CCK-8) and EdU proliferation assays. Wound healing assays and Transwell assays were used to measure the ability of cell migration and invasion. Flow cytometry was used to investigate the effect of *ABCA12* on the proliferation cycle and apoptosis of pancreatic cancer. Western blot analysis detected changes in apoptosis, migration, and other pathway proteins in SW1990 cells after transfection.

**Results:** *ABCA12* is highly expressed in pancreatic cancer tissues and cells. After *ABCA12* was knocked down, the proliferation, invasion, and migration of SW1990 cells were significantly reduced, and apoptosis was increased. The changes in pathway proteins suggested that *ABCA12* may regulate the progression of pancreatic cancer through the AKT pathway.

**Conclusion:** We found that *ABCA12* is differentially expressed in pancreatic tissues and cells. *ABCA12* can also affect the biological behavior of pancreatic cancer cells effectively, which may serve as a new target for pancreatic cancer diagnosis and treatment.

## Introduction

Pancreatic cancer is a common gastrointestinal tumor and a malignant tumor with five-year survival rate less than 8% (Ansari et al., 2016; McGuigan et al., 2018). Healthy lifestyle habits such as avoiding alcohol and having regular exercise can lower the risk of pancreatic disease and even pancreatic cancer (Rawla et al., 2019). The proportion of male patients is higher than female patients. Most of the patients are over 40 years old, but in recent years, the number of patients diagnosed with pancreatic cancer has become younger in China (Lin et al., 2015). The diagnosis of pancreatic cancer is accompanied by atypical early symptoms, such as epigastric discomfort, fever and intermittent diarrhea (Evans et al., 2014). Therefore, the diagnosis of patients got delayed and the best timing of treatment got missed (Wang et al., 2020). Although great advances have been made in the diagnosis and treatment of pancreatic cancer, biomarkers with high specificity are still needed at the micro level. The genesis and development of pancreatic cancer at the molecular level need to be further studied, and new tumor markers need to be discovered, becoming new targets for the early diagnosis and treatment of pancreatic cancer.

*ABCA12* is derived from the huge transporter family ABC (Srikant, 2020), which is expressed in numerous tissues in the human body, such as pancreas, reproductive organs, placenta, lung, heart, intestine, etc., (Davidson and Chen, 2004). *ABCA12* is a transmembrane transporter to transport lipids, including ceramides (Dean et al., 2001). *ABCA12* gene mutation has been confirmed to be associated with three kinds of autosomal recessive congenital ichthyosis (Villanueva et al., 2019), which leads to lipid barrier malformation due to abnormal lipid transport in cuticle cell lamellar particles (Akiyama, 2014). One review showed that *ABCA12* regulates insulin secretion by islet B cells as a lipid transporter (Akiyama, 2010). At present, *ABCA12* is highly expressed in some tumors, but there are few reports on its mechanism of tumor development. In this study, the expression of *ABCA12* in the tumor database was analyzed by bioinformatics and predicted from multiple perspectives. At the same time, the effect of *ABCA12* in pancreatic cancer cells was explored at the clinical tissue level and cell lines constructed, and the AKT pathway was confirmed to facilitate the occurrence of pancreatic cancer.

## Materials and Methods

### Bioinformatics Analysis

The gene expression matrix and clinical information of pancreatic cancer patient samples were obtained from TCGA database (https://www.cancer.gov/about-nci/organization/ccg/research/structural-genomics/tcga). Differences in gene expression matrix analysis by edge2 package in R. Survival analysis of *ABCA12* use Survival package in R (https://www.bioconductor.org. Coexpressed genes of *ABCA12* were obtained from the UALCAN website (https://ualcan.path.uab.edu/index.html). The volcano map of difference analysis, risk prediction model andenrichment analysis of GO pathway were all drawn from Sangerbox (https://www.sangerbox.com), an online analysis website.

### Clinical Samples

From 2013 to 2017, 30 patients with pancreatic cancer and their adjacent normal tissues were collected from Shengjing Hospital affiliated with China Medical University. These patients were not given chemotherapy or radiation before surgery. The study was ratified by the ethics Committee of Shengjing Hospital affiliated to China Medical University after review.

### Immunohistochemistry

Pancreatic cancer and adjacent pathological tissues were dewaxed for 2 h and 40 min, and then the tissues were washed with PBS 3 times for 5 min each. After soaking the tissues in citric acid buffer (ph9.0), heat it in the microwave on high for 7 min and 30 s for Antigen retrieval. Cool to room temperature and wash with PBS as above. Hydrogen peroxide was dropped onto the tissue for 40 min to block endogenous peroxidase activity. After washing with PBS, the slices were incubated with 10% goat serum for 40 min. Monoclonal rabbit anti-*ABCA12* antibody (1:200; PA5-42400; Thremo) was incubated at 4°C overnight. The following day the tissues were restored to room temperature and washed with PBS. Goat anti-rabbit secondary antibody binds to tissues for 25 min and then incubated with streptavidin-peroxidase for another 25 min. Rinse with PBS and DAB staining solution (DAB-0031; Fuzhou Mai Xin) staining sections, distilled water stopped color, and hematoxylin restained the nuclei. After the neutral gum sealing cover slides, the microscope was used for observation, and random photos were taken.

### Cell Culture

HPDE6-C7, HPC-Y5, SW 1990, CAPAN-2 and PANC-A were all from ATCC. All cells were cultured in DMEM containing 10% FBS at 37°C and 5% CO_2_, while PANC-A cells required the addition of 1% sodium pyruvate to the medium.

### Cell Transfection

To silence the expression of *ABCA12*, three siRNases (GIMA Corporation) were used to knock down *ABCA12*. For transfection, SW1990 cells were cultured in 6-well plates (1 × 10^5^/well), 1.2 µg siRNA and 4.5 μL PolyFast Transfection Reagent (Cat. No.HY-K1014; MCE), mix and let stand for 15 min before adding. The cells continued to be cultured at 37°C and 5% CO_2_, and the complete medium was replaced at 6 h. The cells were collected for total RNA extraction at 48 h and total protein extraction at 72 h. RT–qPCR and western blot assays were used to verify whether *ABCA12* is knocked down successfully. All primer sequences are listed in Table 1.

**TABLE 1:** Primer sequences of *ABCA12* and siRNAs.

Name	Sequence
*ABCA12*	Sense:5′-TGACAAGAGGAGAGAGGCTGGATG-3′
	Antisense:5′-TGGCAGTGGTAACAAAGACGATGG-3′
GAPDH	Sense:5′-GTCCCAAACCTTCTGGATCTCTAC-3′
	Antisense:5′-GTCCCAAACCTTCTGGATCTCTAC-3′
si-NC	Sense:5′-UCCUCCGAACGUGUCACGUTT-3′
	Antisense:5′-ACGUGACACGUUCGGAGAATT-3′
si1-*ABCA12*	Sense:5′-CCAGAAGUCUGUUAAACAUTT-3′
	Antisense:5′-AUGUUUAACAGACUUCUGGTT-3′
si2-*ABCA12*	Sense:5′-CCAGGUCAGUUACUAGAAATT-3′
	Antisense:5′-UUUCUAGUAACUGACCUGGTT-3′
si3-*ABCA12*	Sense:5′-GGACAGACCUUAUCUCCAATT-3′
	Antisense:5′-UUGGAGAUAAGGUCUGUCCTT-3′

### RT–qPCR Assay

Total RNA was extracted from HPC-Y5, HPDE6-C7, SW1990, CAPAN-2, PANC-A by using TRIzol reagent (Invitrogen, Thermo Fisher, United States). Total RNA was reverse transcribed into cDNA using the SYBR® Premix Ex TaqTM II (Tli RNaseH Plus) (TaKaRa, Japan) RT–PCR kit. Quantitative PCR was performed on a Roche480 real-time PCR system.

The relative expression of *ABCA12* in five cell lines was calculated by the 2- δ δ Cq method. The experiment was repeated 3 times using different RNAs from three generations of cells.

### CCK-8 Assay

SW1990 cells transfected 48 h later were inoculated into a 96-well plate (2000 cells/well). Each treatment group was set up with three subwells and incubated with 5% CO_2_ at 37°C. After cell adherence, CCK-8 reagent was mixed with complete medium, and added to 96-well plate. Absorbance at 450 nm was detected after 2 h of incubation away from light and was detected at the same time on days 2, 3, 4, and 5. The experiment was repeated three times.

### EdU Assay

SW1990 cells transfected for 48 h were seeded in 96-well plates (10 cells/well × 10^4^ cells/well). After cell adherence, 100 μM 2X EdU was added to each well for 2 h. After washing with PBS, 100 μL of general tissue fixative was added and fixed for 15 min. After washing, cells were lysed with 0.3% Triton X for 15 min. After cleaning, fluorescent staining was added to stain the cells for 30 min. After washing with PBS, Hoechst 33342 was added to each well to stain the nuclei. The experiment was repeated three times.

### Transwell Assay

SW1990 cells were starved 48 h after transfection for 24 h in advance, digested with trypsin, suspended in 200 μL serum-free DMEM. The cell suspension was placed in the transfer chamber (1 cells/well × 10^5^ cells/well). The invasion assay required a layer of matrigengel at the bottom of the cells in advance, while the migration assay directly added cell suspension. Then, 500 μL complete medium containing 10% FBS was added to the lower chamber of the transfer chamber. After incubation for 12–16 h, the cells without transfer were cleaned with PBS twice, and the untransferred cells were wiped with cotton swabs. The cells were fixed with universal tissue fixative for 2 min, methanol infiltration for 20 min, crystal violet staining at room temperature to avoid light for 15 min, washed with PBS and dried with cotton swabs. The number of cells in five fields was recorded at random under a 200X light microscope. The experiment was repeated three times.

### Wound-Healing Assay

SW1990 cells transfected for 48 h were inoculated into a six-well plate (1 cells/well × 10^5^ cells/well) and cultured at 37°Cand 5% CO_2_ until the cells covered the entire six-well plate. Vertical scratches were made on the cell surface with the tip of a 250 μL pipetting gun, and the suspended cells were gently washed with PBS. Serum-free DMEM was replaced, and culture was continued. The field of vision at the same position in each hole was photographed at 0 and 48 h. The distance of scratches on both sides was used to reflect the migration ability of cells. The experiment was repeated three times.

### Flow Cytometry

Cell cycle and apoptosis were measured by flow cytometry. During the detection cycle, cells were digested with EDTA-free trypsin and centrifuged at 1200 r/min, and the supernatant was discarded. The cells were resuspended in 70% alcohol and centrifuged at 1200 r/min after washed by PBS. Finally, cell precipitates were stained with propidium iodide (PI, 500 μL/tube) in a flow cytometry tube for 15 min, and cell cycle levels were analyzed by flow cytometry. For the detection of cell apoptosis, and 100 μL banding buffer was added to resuscitate the cells. PI and junctin V-FITC were used to avoid light staining for 10 min. Another 400 μL banding buffer was added to each flow tube to terminate the reaction. Data were collected using flow cytometry. The above operations were repeated three times.

### Western Blot Analysis

Seventy-two hours after transfection, HPC-Y5, HPDE6-C7, SW 1990, CAPAN-2 were washed with phosphate buffered saline (PBS). The cells were lysed with RIPA lysis buffer (Beyotime Biotechnology Institute). Add 1X LoadingBuffer in a ratio of 4:1, boil for 5 min at 100°C, cool and store at −80°C for later use. Proteins were isolated by 10% SDS-PAGE (15 µg/swim lane) and transferred to 0.45 µM PVDF membranes (IPVH00010, Millipore). Seal the membrane with 5% skim milk at 37°C for 2 h. A Tris buffer salt solution containing 0.1% Tween-20 (TBST) was prepared. TBST membranes were washed 3 times and incubated overnight at 4°C: monoclonal antibodies *ABCA12* (1:1500; PA5-42400; Thremo), anti-BCl2 (1:2000; Cat No: 12789-1-AP; Proteintech), anti-BAX (1:4000; 50599-2-Ig; Proteintech), anti-Caspase-3 (1:1000; T40044; Abmart), anti-cleaved-Caspase-3 (1:1000; 9661S; Cell Signaling Technology), anti-MMP2 (1:2000; Cat No: 10373-2-AP; Proteintech), anti-MMP9 (1:1000, Cat No: 10375-2-AP; Proteintech), anti-AKT (1:1,000; Ab179463; Proteintech), anti-P-AKT (1:1,000; ab38449; Abcam), and anti-PI3K (1:1,000; ab38449; Abcam). After returning to room temperature the next day, the membrane was washed by TBST3 and mixed with goat anti-rabbit immunoglobulin (1:10000; Cat# AF7021; Affinity) at 37°C for 2 h. After washing with TBST, Beyotime Institute of Biotechnology (ECL) would stimulate the protein bands to verify the differential expression of the five cell lines and detect the knockdown efficiency of si1-*ABCA12* and si3-*ABCA12*. Changes in apoptosis, metastasis and pathway-related protein expression after knockdown. The experiment was repeated three times for each index.

### Statistical Analysis

GraphPad Prism 6 was used for image editing in this study. SPSS 20.0 was used for statistical analysis of clinical data. *T* test was used to compare the differences between the two groups, and one-way ANOVA/two-way ANOVA was used to compare and analyze the data between multiple groups. Data are presented as the mean ± standard error. *p* value less than 0.05 represents a significant difference. Each experiment was repeated under the same conditions.

## Results

### *ABCA12* Expression in Pancreatic Tissues was Analyzed Based on Biological Information Base

**FIGURE 1:**
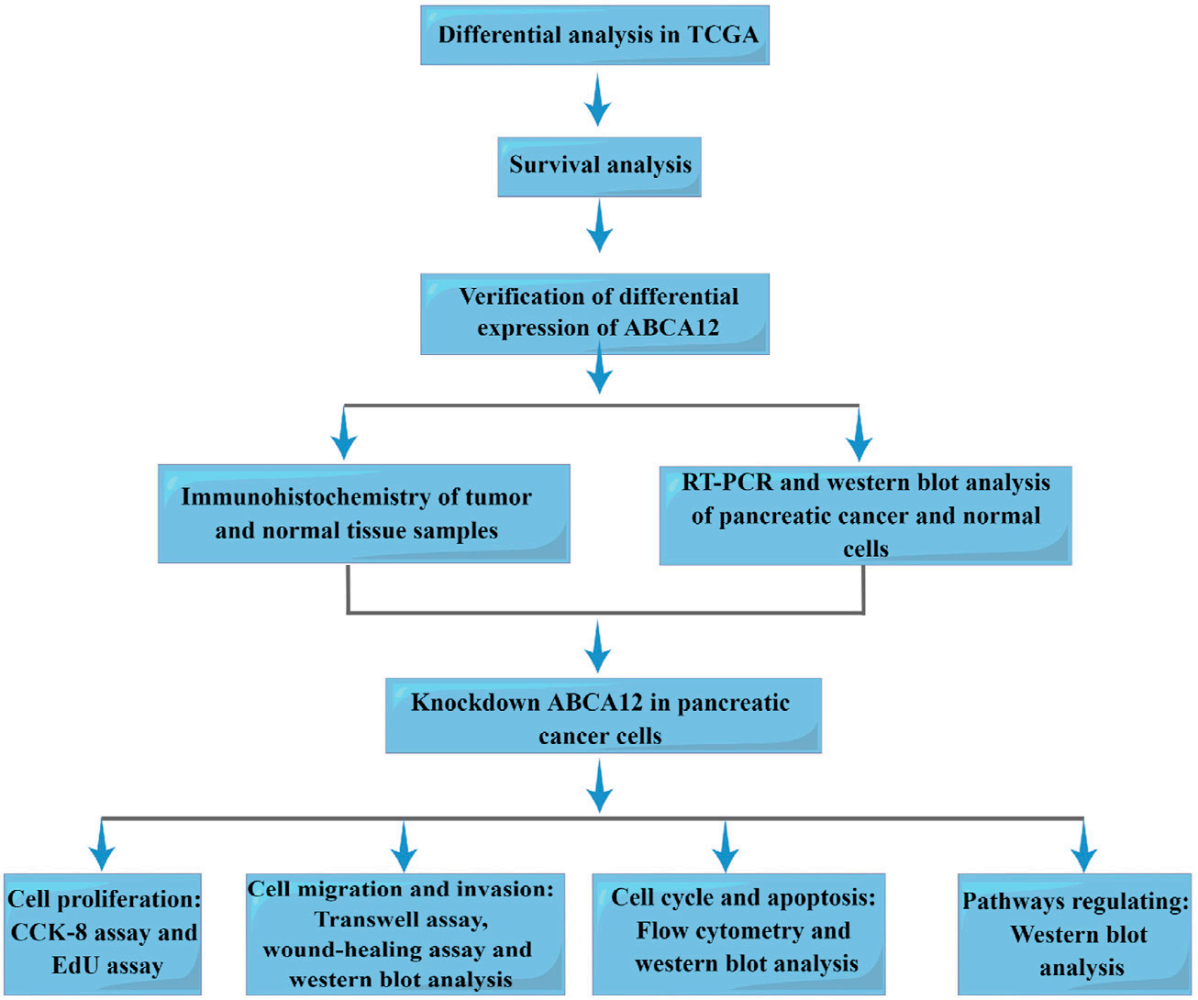
Workflow of this study.

**FIGURE 2:**
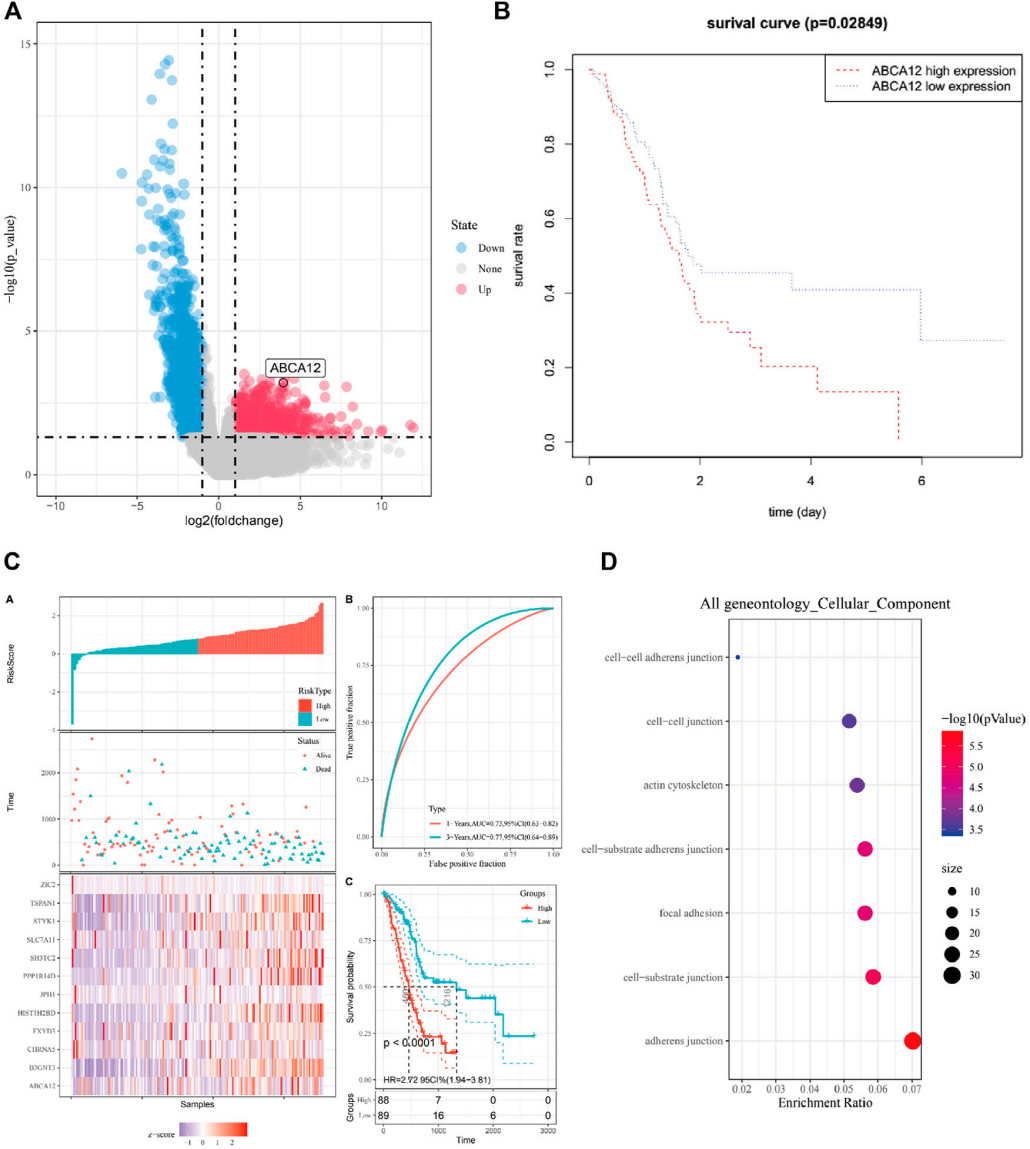
Bioinformatics analysis of *ABCA12*. **(A)** Volcano map of differential genes in TCGA pancreatic cancer database. **(B)** Survival curve of patients expressing *ABCA12* in TCGA database. **(C)** Prediction model of risk score for highly expressed genes in TCGA database. **(D)** Bubble map of GO enrichment analysis of *ABCA12* coexpressed genes in TCGA pancreatic cancer database.

Figure 1 is the workflow chart of the whole study. In the TCGA database, gene expression differences between pancreatic cancer samples and normal samples were analyzed and visualized by volcano map. *ABCA12* was a highly expressed gene with a multiple of more than 2 times, and the difference was statistically significant (Figure 2A). Univariate survival analysis of the *ABCA12* gene showed that the five-year survival rate of patients with high *ABCA12* expression was obviously lower than control group (*p* = 0.02849 < 0.05) (Figure 2B). The survival curves of other highly expressed genes were not statistically significant or had been reported and studied in the literature (Supplementary Figure S1). Risk prediction was performed on TCGA database samples, and heat map of differential genes and patient prognosis scores were drawn. The results demonstrated that populations with highly expressed genes, including *ABCA12*, possessed a higher risk, suggesting poor prognosis (Figure 2C). In the UALCAN online website (https://ualcan.path.uab.edu), access to TCGA pancreatic cancer database with *ABCA12* expressed genes go analysis, through the bubble chart presents the group of genes tend to cell adhesion function (Figure 2D). The above illustration was made *via* sangerbox (https://www.sangerbox.com).

### *ABCA12* is Upregulated in Pancreatic Cancer Tissues and Cell Lines and is Successfully Knocked Down by siRNAs

**FIGURE 3:**
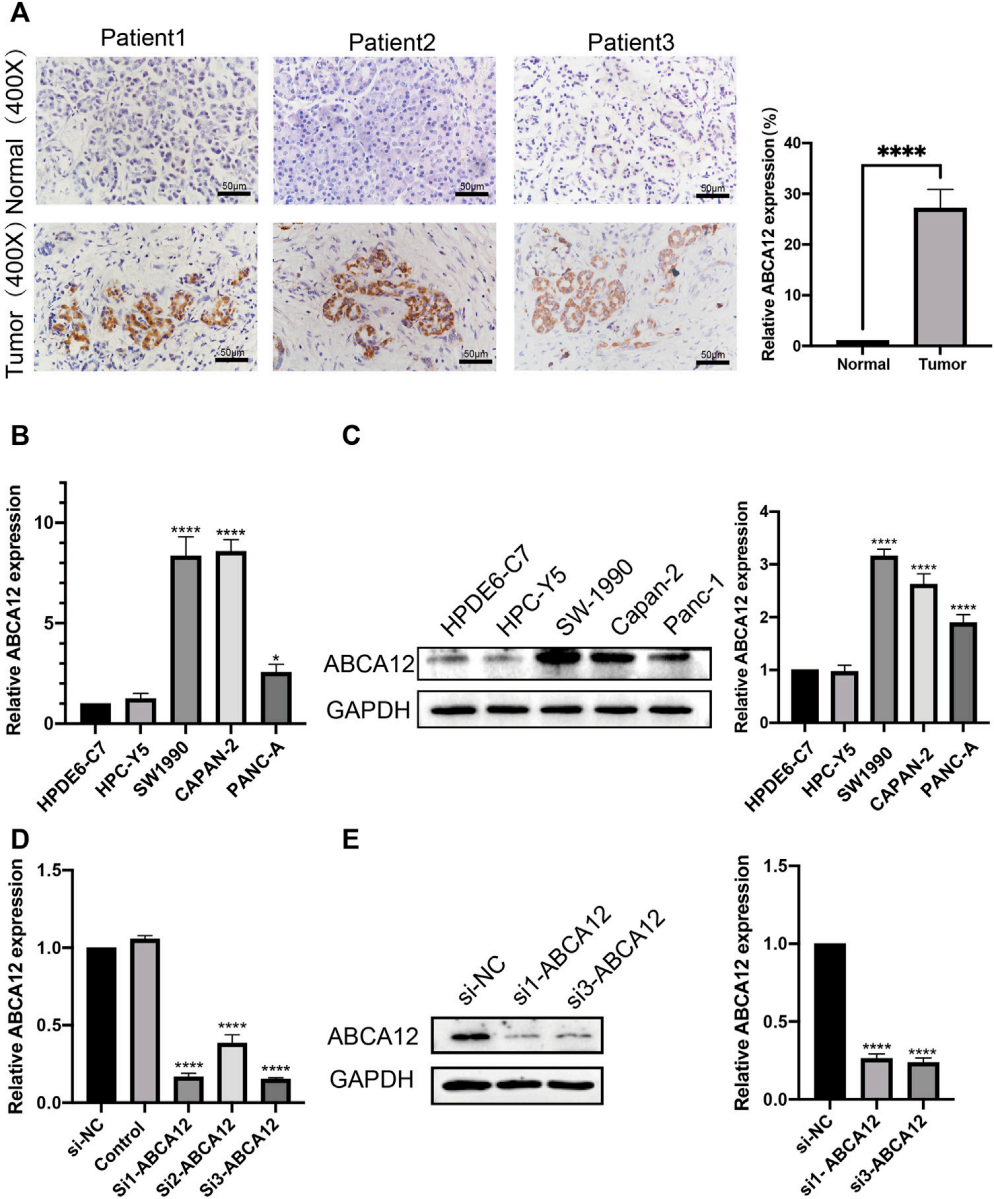
mRNA and protein expression of *ABCA12* in pancreatic cancer. **(A)** IHC results manifested that *ABCA12* was highly expressed in pancreatic cancer tissues. **(B,C)** The expression of *ABCA12* mRNA and *ABCA12* protein in pancreatic cancer cells was higher than that in normal pancreatic cells. **(D,E)** *ABCA12* in SW1990 cells was effectively knocked down by siRNA, and si1-*ABCA12* and si3-*ABCA12* had the best knockdown efficiency.

The expression of *ABCA12* in cancer and paracancerous tissues of 30 clinical patients was verified by immunohistochemical experiments. The results pointed out that *ABCA12* was highly expressed in the part of the pancreatic duct, while *ABCA12* was significantly expressed low in normal pancreatic cells of paracancerous tissues (Figure 3A). All pancreatic cancer tissues were shown to contain no expression of AFP protein (Supplementary Figure S2). The relationship between *ABCA12* expression and clinical information in pancreatic cancer tissues of 30 patients wss shown in Table 2. High expression of *ABCA12* was closely related to tumor grade and lymphnodes. RT-PCR and immunoprotein imprinting assays were supposed to testify the mRNA and protein expression of *ABCA12* in five cell lines. Compared with the normal pancreatic cell lines HPC-Y5 and HPDE6-C7, SW1990, CAPAN-2 and PANC-A showed significant upregulation at the mRNA level and the same trend at the protein level (Figures 3B,C). The SW1990 cell line was the most obviously upregulated at both levels. Three siRNAs with different sequences were used to knock down the *ABCA12* in SW-1990 cells, and si1-*ABCA12* and si3-*ABCA12* had more obvious knockout efficiency than si2-*ABCA12* (Figures 3D,E). The results proved that *ABCA12* is significantly upregulated in pancreatic cancer cells and that si1-*ABCA12* and si3-*ABCA12* have the most significant inhibitory effects on *ABCA12*.

**TABLE 2:** Relative expression of *ABCA12* and clinical features of patients.

Variable	Groups	Total	High	Low	*p*
Age (years)	<60	13	6	7	0.31
	≥60	17	11	6	
Gender	Male	19	11	8	0.86
	Female	11	6	5	
Tumor size (cm)	≥3	22	12	10	0.7
	<3	8	5	3	
Tumor grade	I/II	22	10	12	0.04*
	III/IV	8	7	1	
Tumor stage	T1+T2	23	11	12	0.08
	T3+T4	7	6	1	
Lymphnodes	Present (N1–N3)	8	7	1	0.04*
	Absent (N0)	22	10	12	
Distant metastasis	Present (M1)	2	2	0	0.20
	Absent (M0)	28	15	13	

### After *ABCA12* Knockdown, the Proliferation and Metastasis of SW1990 Cells Were Weakened, and the Apoptosis Level was Increased

**FIGURE 4:**
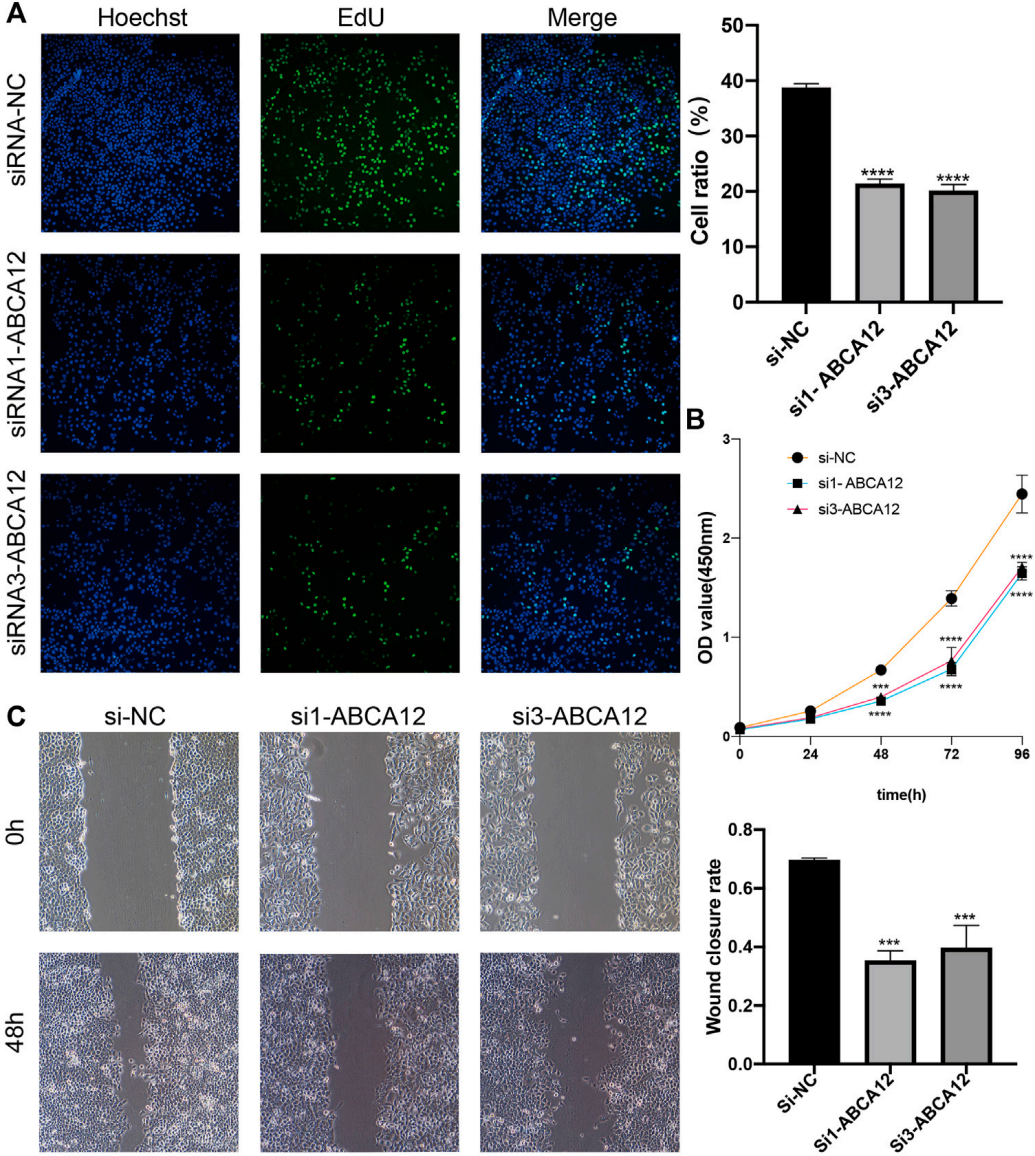
Changes in proliferation and migration of SW1990 cells after *ABCA12* knockdown. **(A)** EdU experiments showed that the proliferation ability of pancreatic cancer cells was significantly reduced after *ABCA12* knockdown. **(B)** CCK-8 assay showed that the proliferation ability of pancreatic cancer cells was reduced after *ABCA12* knockdown. **(C)** Scratch healing experiments showed that the migration ability of pancreatic cancer cells was significantly reduced after *ABCA12* knockdown.

**FIGURE 5:**
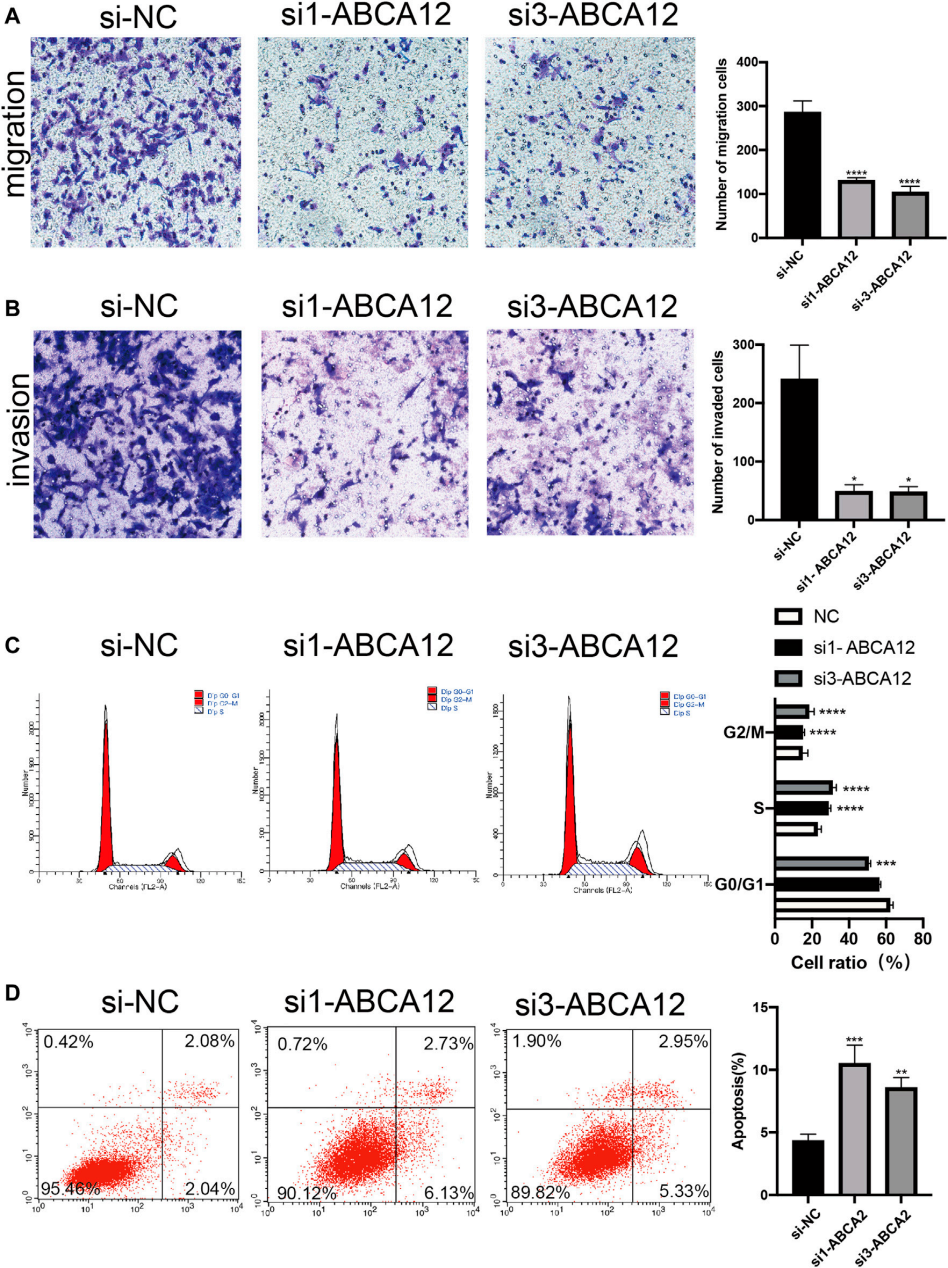
Transwell and flow cytometry of SW1990 cells after *ABCA12* knockdown. **(A)** Transwell assays showed that *ABCA12* knockdown reduced the migration capacity of pancreatic cancer cells. **(B)** Transwell assays with marigel proved that the invasion ability of the knockout group was decreased. **(C)** Flow cytometry showed that the number of G0/G1 phase cells decreased and that of S phase cells increased after *ABCA12* was inhibited. **(D)** Flow cytometry showed that the apoptosis level was significantly increased after *ABCA12* was knocked down.

EdU proliferation assays demonstrated that amount of proliferating cells in the si1-*ABCA12* and si3-*ABCA12* knockout groups was less than that in the negative control group, indicating a reduced proliferation level (Figure 4A). The CCK-8 assay manifested that compared with the negative control group, the proliferation activity of SW1990 cells was reduced after si1-*ABCA12* and si3-*ABCA12* were used to knock down *ABCA12* (Figure 4B). In the scratch healing experiment, the healing area of *ABCA12* knockdown cells increased significantly 48 h later (Figure 4C), and the number of cells crossing the subtractal compartment in the Transwell experiment decreased significantly after *ABCA12* knockdown, suggesting that the metastatic and invasion ability of pancreatic cancer cells was weakened after *ABCA12* silencing (Figures 5A,B). Flow cytometry showed that *ABCA12* knockdown reduced the proportion of G0/G1 phase cells and significantly increased the proportion of S phase cells, suggesting that *ABCA12* knockdown mainly caused the arrest of pancreatic cancer cell proliferation by inhibiting S phase (Figure 5C). Apoptosis assays proved that the total ratio of early apoptosis and late apoptosis was significantly increased after *ABCA12* was knocked out, indicating that *ABCA12* inhibited apoptosis (Figure 5D). After the addition of gemcitabine, the results showed that the apoptosis rate of the treatment group was higher than that of the target genome knockout group, while the apoptosis rate of the common processing group was the highest (Supplementary Figure S3). This suggest that *ABCA12* knockdown can assist in the co-promotion of tumor cell apoptosis by therapeutic drugs. Therefore, *ABCA12* could promote the proliferation, metastasis and antiapoptosis of pancreatic cancer cells.

### Western Blot Assays Were Performed to Detect Apoptosis/Metastasis/Pathway-Related Proteins

**FIGURE 6:**
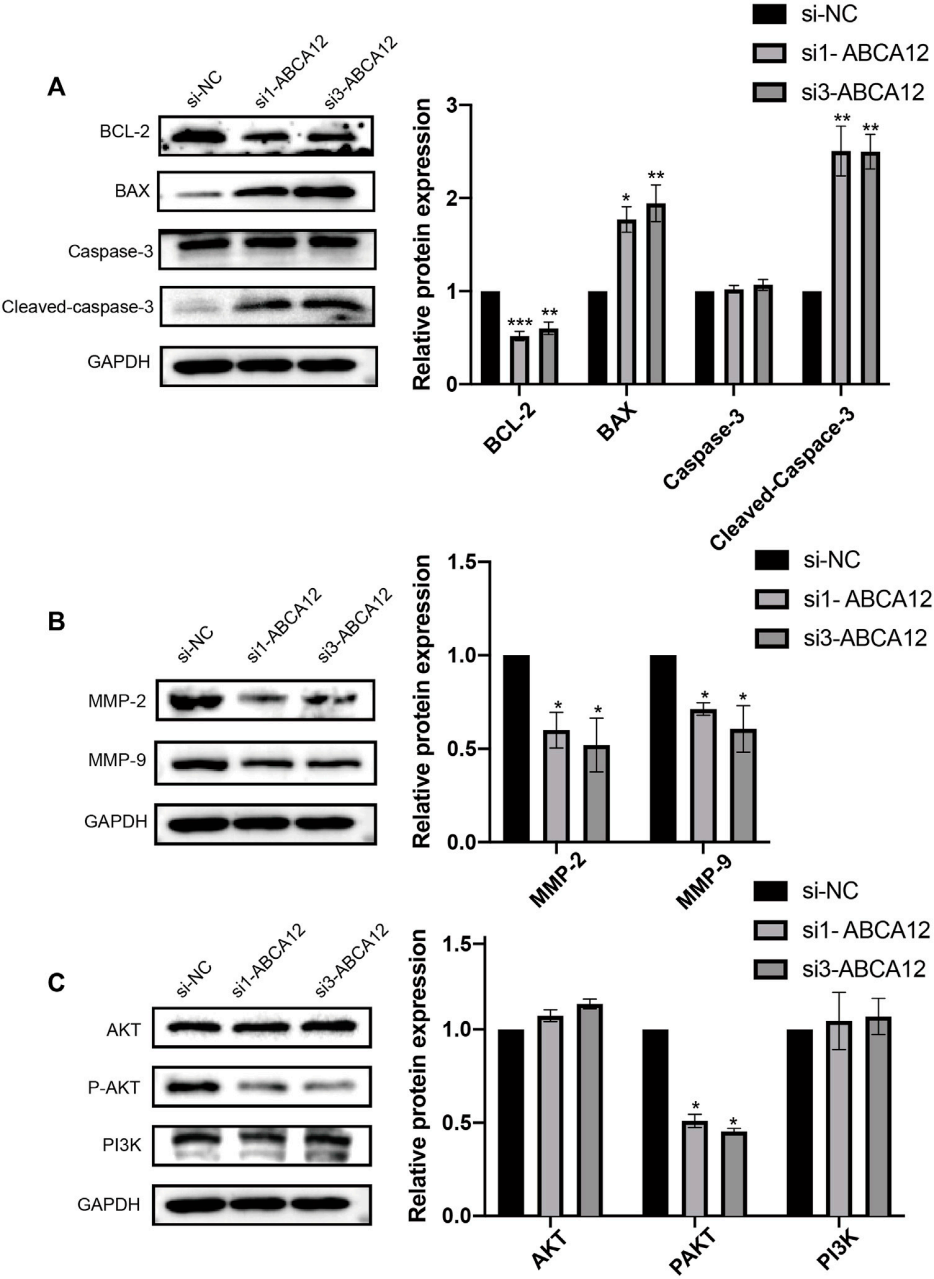
Changes of apoptosis, migration and pathway proteins in SW1990 cells after *ABCA12* knockdown. **(A)** After *ABCA12* knockdown, the expression level of BCL2 protein was decreased, and BAX protein was increased. Cleaved caspase3 expression was decreased when caspase3 remained unchanged. **(B)** The expression of MMP-2 and MMP-9 was significantly decreased after *ABCA12* knockout. **(C)** After *ABCA12* inhibition, the expression of P-AKT decreased, while the expression of AKT and PI3K remained basically unchanged.

Western blot assays were used to detect cell functional proteins, and the expression of the antiapoptotic protein BCL2 was decreased in the *ABCA12* knockout group and higher in the negative control group. The expression of the apoptotic protein BAX in *ABCA12*-silenced cells was significantly higher than that in the negative control group. Cleaved-Caspase3 expression was significantly increased in the knockdown group on the premise that Caspase3 protein expression was unchanged in each group (Figure 6A). For the detection of transfer proteins MMP2 and MMP9, the expression of *ABCA12* cells was significantly lower than that of the corresponding protein in the negative control group (Figure 6B).

Pathway proteins in *ABCA12* knockdown cells and negative control cells were detected. Immunoprotein imprinting test results showed that the expression of AKT and PI3K proteins did not change significantly in the two groups of cells. The expression of phosphorylated P-AKT in *ABCA12* knockout cells was significantly lower than that in negative control cells (Figure 6C). Therefore, *ABCA12* induces the occurrence and development of pancreatic cancer through the AKT pathway.

## Conclusion

In this study, we obtained the gene expression matrix of 182 patients with pancreatic cancer from the TCGA database and confirmed the differential expression of *ABCA12* in pancreatic cancer and para-cancer samples. Survival analysis results showed that the five-year survival rate of patients with high expression of *ABCA12* was significantly lower than that of the control group. Meanwhile, we demonstrated the over-expression of *ABCA12* in pancreatic cancer at the cellular level and in clinical tissues for the first time. Through functional tests, we found that the proliferation and metastasis of pancreatic cancer cells with *ABCA12* knockdown were significantly decreased, and the apoptosis level was significantly increased. Further experiments demonstrated that *ABCA12* promoted the proliferation and metastasis of pancreatic cancer by activating the AKT pathway and effectively inhibited apoptosis, which provided a new direction in molecular-level research on pancreatic cancer and new ideas for future treatment strategies for pancreatic cancer.

Worldwide, pancreatic cancer is the seventh leading cause of cancer death precisely (Scott et al., 2013). According to research, pancreatic cancer will be the second leading cause of cancer death in the United States by 2030 (Ursino et al., 2020). Adenocarcinoma is the pathologic type of 90% of pancreatic cancers (Khalaf et al., 2021). The incidence and mortality of pancreatic cancer is on the rise recently, which severely threatens health of people worldwide (Rahib et al., 2014). On the one hand, pancreatic cancer is characterized by strong metastasis and poor prognosis, which can be extremely destructive to the primary organ of the tumor or to the tissue after metastasis. On the other hand, its onset is relatively insidious, and patients have no specific clinical characteristics, so that often consuming the best time for diagnosis and treatment. Therefore, through the study of pathogenesis, it is in urgent need of finding new markers. Some researches indicate that PMEPA1 inhibits the proliferation, invasion and migration of pancreatic cancer cells by activating the PTEN/PI3K/AKT pathway (Goral, 2015). TRPM2 promotes pancreatic cancer through the PKC/MAPK pathway (Wu et al., 2018). Pin1 promotes pancreatic cancer progression and metastasis by activating the NF-κB-IL-18 feedback loop (Yang et al., 2021). A growing number of proteins are affirmed to be involved in the occurrence of pancreatic cancer. In this study, we focused on the study of *ABCA12* in the pathogenesis of pancreatic cancer and analyzed its carcinogenic mechanism from multiple perspectives through cell line functional phenotypes, clinical tissue data and database information. *ABCA12* may provide a brand-new target for pancreatic therapy.

ABC is a huge superfamily of transporters, mainly divided into 7 subfamilies (Lin et al., 2021). It was first discovered in the study of bacterial nutrient metabolism in 1970 (Sun et al., 2020). It has a variety of biological functions and supports organisms in completing many key physiological processes. In human body, most ABC proteins transport compounds from the cytoplasmic side to the outside of the cell membrane to complete transport and *in vivo* secretion (Liu, 2019). ABC family proteins are often mentioned in studies on tumor drug resistance (Nobili et al., 2020). Some proteins in the family have been proved to be related to the occurrence of some specific diseases. ABCA1/ABCA7 is bound up with Alzheimer’s disease, ABCA4 is in connection with Stargardt macular degeneration, and ABCC8/SUR1 and ABCC9/SUR2 are both associated with diabetes (Theodoulou and Kerr, 2015). *ABCA12* is a member of the ABC1 subfamily, the only group found in multicellular eukaryotes (Schmitz and Langmann, 2001). *ABCA12* is widely expressed in skin tissues, and this gene is known to be derived from numerous literature reports on congenital ichthyosis Harlequin, which can promote the formation of neurolinoleyl ester to maintain the infiltration barrier of epidermal lipids (Glick et al., 2017). Mutation of *ABCA12* leads to defects in lipid transport and dysfunction of the skin barrier, leading to congenital ichthyosis (Yamanaka et al., 2007). In addition, differential expression of *ABCA12* in metastatic advanced Egyptian bladder cancer has been reported in the tumor field (Zekri et al., 2015). Some studies screened out some repeatedly mutated genes, including *ABCA12*, through cancer genome maps and RNA sequencing data as new markers of colorectal adenocarcinoma (Wang et al., 2021). High expression of genes, including *ABCA12*, was also detected in an army of 151 cancer sufferers (breast cancer, colorectal cancer, and pancreatic cancer) (Dvorak et al., 2017). At present, the progress of the ABCA subfamily in tumor research has been further revealed and studied, and it is speculated that it has a certain risk for tumor development and prognosis (Pasello et al., 2020). However, there is still no relevant research on the occurrence and mechanism regulation of tumors, and *ABCA12* has never been studied on the mechanism and function of tumors.

AKT is a representative signaling channel that existed in the human body that regulates glucose homeostasis, protein synthesis, and transport, cell proliferation, migration, inhibition of apoptosis, vesicle transport, etc., (Xie et al., 2019). This pathway can trigger tumor growth, leading to angiogenesis due to abnormal stimulation (Jiang et al., 2020). The impact of the AKT pathway in regulating T cell development, operation, and stability has been reported (Pompura and Dominguez-Villar, 2018). To date, the AKT pathway has been well studied in the molecular mechanism of malignant tumors. It has been documented that inhibition of this pathway provides a possibility for addressing tumor drug resistance, and pathway inhibitors can improve the receptance of tumor cells to drug-induced apoptosis (Liu et al., 2020). Studies have shown that the activation of the PI3K pathway due to *PTEN* mutation leads to the occurrence of primary prostate cancer (Yan and Huang, 2019). In colorectal cancer, *GF2* overexpression, *PIK3CA* mutation, *PTEN* mutation, and deletion can open the PI3K/AKT pathway (Danielsen et al., 2015). After inhibiting the PI3K/AKT signaling pathway, it was found in studies on gastric cancer cells that p-PRAS40-Thr246 levels were decreased and apoptosis levels were increased. Cell proliferation and invasion are inhibited (Lu et al., 2019). In pancreatic cancer, *CMTM4*, *LAMB3*, and *IGF2BP2* all promote tumor proliferation, invasion, and metastasis through this activation pathway while inhibiting the cell cycle and apoptosis to play a carcinogenic role (Xu et al., 2019; Zhang et al., 2019; Li et al., 2020). In our study, the western blot assay detected a greater drop in the expression of PAKT in cells after *ABCA12* knockdown, while AKT and PI3K remained basically unchanged, confirming that *ABCA12* plays an essential part in inducing the occurrence and development of pancreatic cancer via this pathway. However, some literatures have speculated that AKT plays an anti-apoptotic role in keratinocytes lacking *ABCA12* expression (Yanagi et al., 2011). *PPAR-δ* has been reported to have an anti-apoptotic effect in keratinocytes by activating AKT1 new pathway through transcriptional regulation (Di-Poï et al., 2002). *PPAR-δ* and *ABCA12* interact in glial cells (Jiang et al., 2008). We surmise that the anti-apoptotic effect of AKT may be due to the regulation of PPAR-δ, and there is a mechanism of interaction between *PPAR-δ* and *ABCA12*. However, it is still unclear how *PPAR-δ* interacts with *ABCA12* in keratinocytes, and whether the upregulation of *PPAR-δ* is a response to apoptosis. In our study, pancreatic tumor cells and keratinocytes may have different differentiation differences, and there may be more gene co-regulation in the internal regulation mechanism of AKT pathway, which needs to be further studied in the future.

In conclusion, the function of *ABCA12* in pancreatic cancer remains to be further revealed. Proteins in the ABC family, including *ABCA12*, have been studied by an increasing number of scientists in the field of cancer, and proteins in this family are expected to become new diagnostic and therapeutic targets in the field of cancer.

## Data Availability

The datasets presented in this study can be found in online repositories. The names of the repository/repositories and accession number(s) can be found below: https://www.jianguoyun.com/c/sd/14ce543/1ad2a2e58c6f32bb.
