# Supplementary figures and images for: ABCA12 Promotes Proliferation and Migration and Inhibits Apoptosis of Pancreatic Cancer Cells Through the AKT Signaling Pathway
Source: Front Genet. 2022 Jun 16;13:906326. doi: 10.3389/fgene.2022.906326 (PMC9243331; doi:10.3389/fgene.2022.906326)

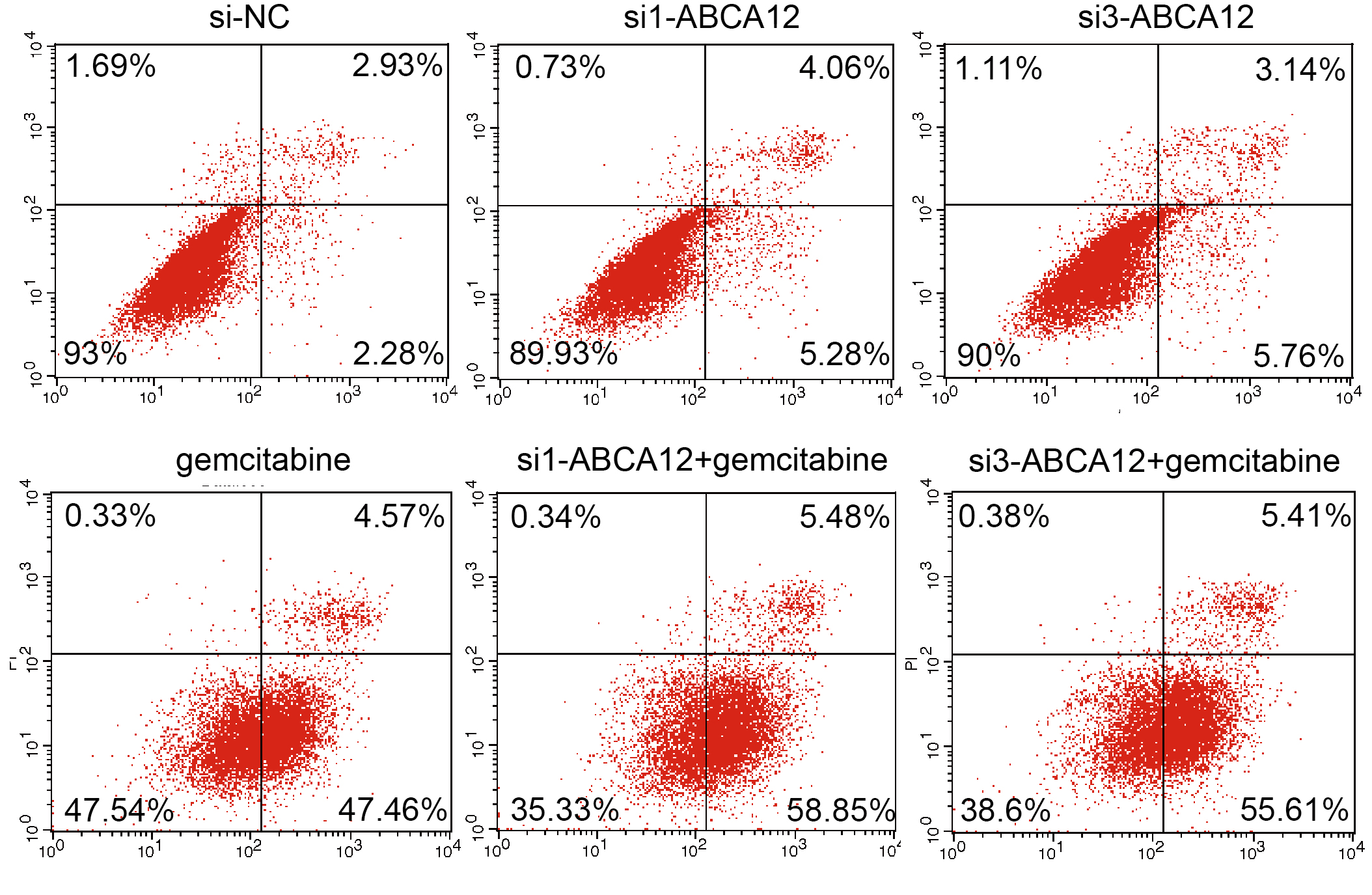

Supplement: Supplementary file 1 [file Image3.JPEG]

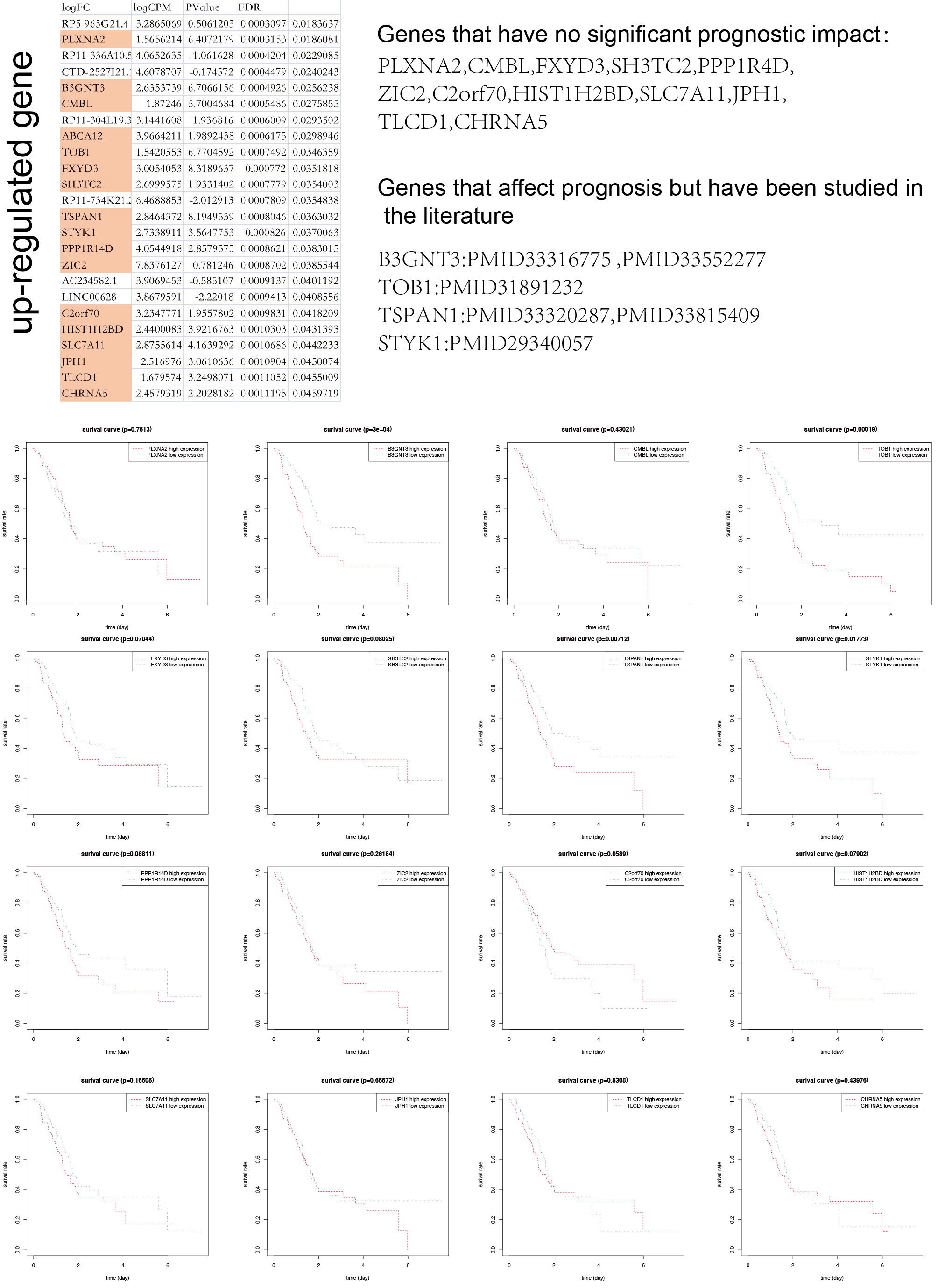

Supplement: Supplementary file 2 [file Image1.JPEG]

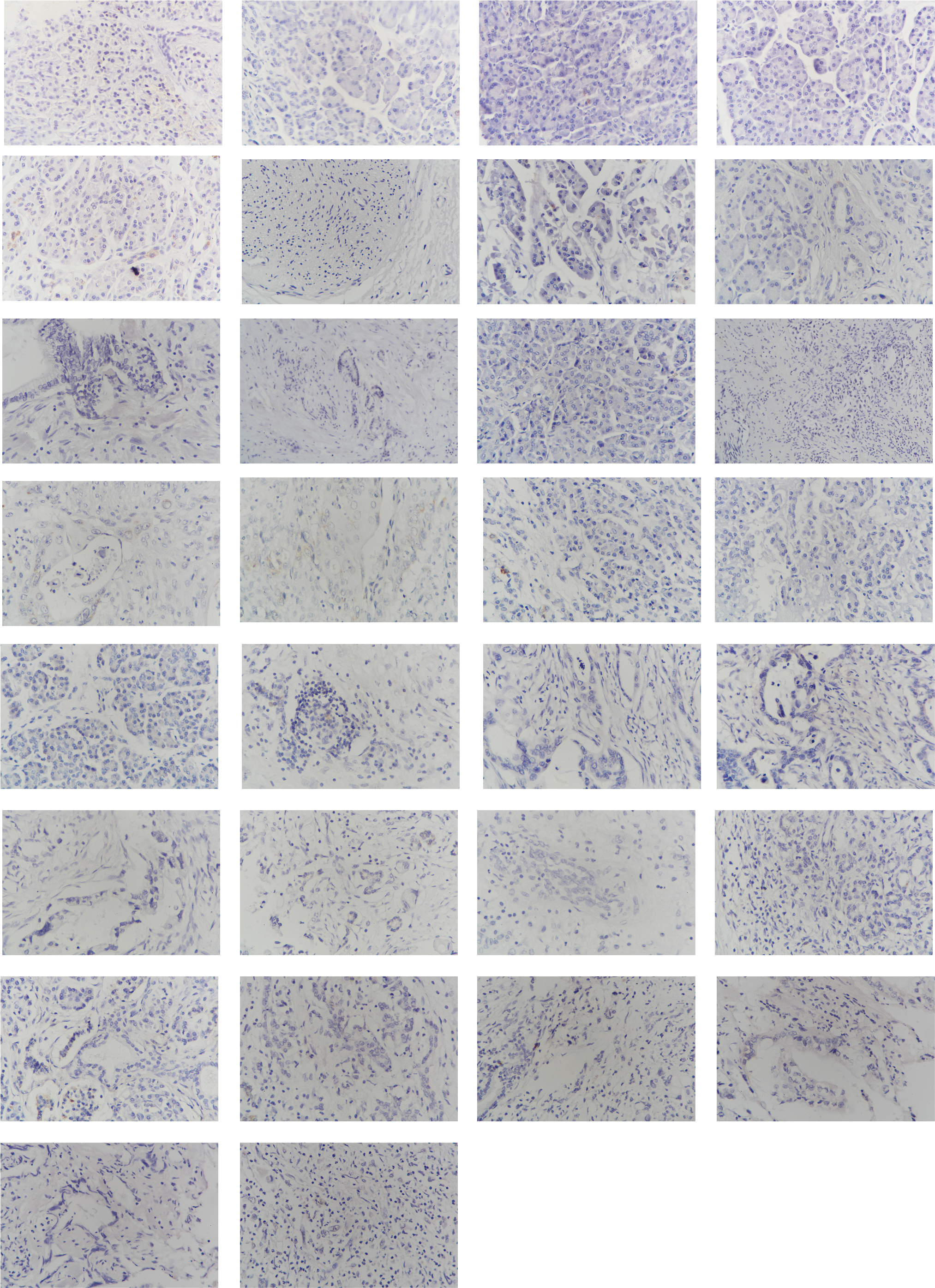

Supplement: Supplementary file 3 [file Image2.JPEG]
